# It Takes a Mouth to Eat and a Nose to Breathe: Abnormal Oral Respiration Affects Neonates' Oral Competence and Systemic Adaptation

**DOI:** 10.1155/2012/207605

**Published:** 2012-07-03

**Authors:** Marie Trabalon, Benoist Schaal

**Affiliations:** ^1^Laboratoire d'Ethologie Animale et Humaine, CNRS (UMR 6552), Université de Rennes 1, 35042 Rennes, France; ^2^Centre des Sciences du Goût et de l'Alimentation, CNRS (UMR 6265), Université de Bourgogne, 21000 Dijon, France

## Abstract

Mammalian, including human, neonates are considered to be obligate nose breathers. When constrained to breathe through their mouth in response to obstructed or closed nasal passages, the effects are pervasive and profound, and sometimes last into adulthood. The present paper briefly surveys neonates' and infants' responses to this atypical mobilisation of the mouth for breathing and focuses on comparisons between human newborns and infants and the neonatal rat model. We present the effects of forced oral breathing on neonatal rats induced by experimental nasal obstruction. We assessed the multilevel consequences on physiological, structural, and behavioural variables, both during and after the obstruction episode. The effects of the compensatory mobilisation of oral resources for breathing are discussed in the light of the adaptive development of oromotor functions.

## 1. Introduction

During the first months of life, mammalian infants are considered to be “obligate nose breathers” [1], although the qualifying term “preferred nose breathers” was proposed subsequently [2]. In any event, these wordings highlight the fact that newborn and very young mammals depend on nasal breathing to adapt their behaviour competently, especially in relation to ingestion and, in newborns, to sucking*—*the specialisation of oral behaviour evolved by mammalian infants in response to mothers having evolved nipples or teats as appendages for milk transfer [3].

While the motor process underlying respiration relies on a centrally controlled automatism, its execution has multiple general consequences, beginning with the most peripheral structures that channel airflow. The resistance of air through the nasal passages has a formative effect on the nasal cavities [4]. Under normal breathing conditions, alternating conchae as well as the nasal cycle within the nose lessens the airflow speed and creates turbulent flow conditions that contribute to shape nasal structures. The nasal inflow also “acclimatises” the physicochemical properties (temperature, hygrometry, and cleanliness through dust adsorption) of incoming air, thus optimising both pulmonary exchanges and chemosensory reception [4–6]. The upper airway crosses the oral path in the larynx region, where respiration and ingestion (and sometimes egestion) are rendered exclusive by the epiglottic switch during feeding. In newborn and suckling infant mammals, both pathways are mobilised serially during ingestive sequences as breathing is compatible with sucking (and later mastication) but not with swallowing [7–10]. Therefore, hazardous aspirations into the lower respiratory pathway are in principle avoided while sucking [11], except during feeding in preterm infants [12]

Beyond ingestion, nose breathing is the dynamic component of olfaction, either in its baseline form through regular inhalation/exhalation or in a specific form characterised by an accelerated rhythm or deeper inhalations, called sniffing. Both inhalation forms create an air flow that carries volatile compounds to intranasal chemosensory nerve endings that give rise to olfaction and trigeminal sensations. Corresponding sensory inputs promote guidance to the offspring to reach the breast, to encode milk/food odour as rewarding (retronasal olfaction), and to monitor the caregiver's odour (orthonasal olfaction) for identity recognition, solace, and energy conservation [13]. Finally, nasal trigeminal sensation caused by the incoming airflow constitutes a regulatory input to the respiratory rhythm generator [14], and its silencing through nasal occlusion may thus subsequently alter respiratory performance.

Nasal breathing is thus multiply related to adaptive oral function, that is, by ensuring an ongoing supply of oxygen during food intake and by supporting olfaction and related motivation to seek the food source and sustain feeding. Thus, any disruption of the normal nasal breathing route through (uni- or bilateral) nasal obstruction may affect not only the respiratory function but also all interdependent sensorimotor nasal, oral, and laryngeal functions. Nasal obstruction can result from either congenital or postnatal causes and may amplify resistance to air-flow and impair sucking-swallowing responses, with increased risks of aspiration or of more severe and threatening respiratory distress conditions [15]. In addition, nasal obstruction alters the “trophic” flow of sensory information towards the olfactory brain. Sensory deprivation due to early nasal obstruction has indeed repeatedly been demonstrated to alter both the structure of animals' olfactory tracts and related functions [16–18].

Here, we present and discuss the immediate and deferred effects of constraining neonatal organisms to breathe through the mouth in response to obstructed or closed nasal passages. A brief survey of human newborns' and young infants' responses to nasal obstruction is paralleled with the results obtained by an experimental approach using neonatal rats. Nasal obstruction was induced experimentally in newborn pups to assess the multilevel consequences on physiological, structural, and behavioural variables, both during and after the obstructive procedure. The effects of respiratory impairment are discussed in light of adaptive development of oromotor functions.

## 2. Impact of Nasal Obstruction in Human Neonates and Infants

### 2.1. Causes of Nasal Obstruction

Natural causes of complete nasal obstruction are rare, but vary in human newborns and infants. The most extreme forms are due to congenital laryngomalacia, bilateral choanal atresia, or oronasal defects associated with Pierre Robin syndrome [19]. Less extreme forms involve choanal stenosis, unilateral choanal atresia, or defects of the nasal septum related to cleft palate [20]. Other mechanical causes such as those due to obstructive tissue masses (adenoid or/and tonsillar hypertrophy) prevail during later development. More benign, short-term obstructive forms derive from mucosal accumulation due to neonatal infections or allergic rhinitis [21–23]. Rhinitis symptoms result from dilation of venous capacitance vessels in the nasal mucosa, mucosal edema, and excess secretions. Allergic rhinitis is very common in infants and children [40% of children are affected in United States; e.g., [24]], as is adenoidal and tonsillar hypertrophy.

Finally, iatrogenic interventions relying on nasogastric tubes or narial tape also have an effect on nasal patency [23]. Inserting nasogastric feeding tubes produces an important (unilateral) increase in nasal airway resistance, thus leading to an increase in respiratory effort [25, 26].

All the above-mentioned causes of obstructive nasal airways can be associated with physiological conditions that may potentiate their effects. First, nasal resistance is greatest during infancy, when airways are narrower [27]. Thus, we can expect that the effects of nasal obstruction would be more important for small-sized bodies, namely, newborns, and even more preterm newborns. Second, nasal patency fluctuates normally between the two nasal cavities by changes in the engorgement of the mucosal vessels in the middle and lower turbinates, a normal variation known as the “nasal cycle.” The magnitude of nasal resistance alternates in each nasal cavity every 2 to 4 h in 60–70% of healthy individuals [28]. Finally, posture can substantially influence the degree of vascular congestion in the nose. Nasal obstruction increases bilaterally as a subject assumes the supine position and increases in the dependent nasal passage in the lateral recumbent position [29].

### 2.2. Short- and Long-Term Impacts on Oral Function

Nasal obstruction forces normal nasal breathing into oral breathing. Numerous clinical observations and experiments show that this apparently benign change has in fact immediate and/or deferred cascading effects on multiple physiological and behavioural functions. First, it has an obvious perturbing impact on newborns' and very young infants' sucking-swallowing activities, and growth is affected accordingly [20]. Nasal obstruction in older infants and children, linked to hypertrophied adenoids or tonsils, is related to growth stagnation, which normalises after surgery [30–32]. It also affects young infants' behaviour, for instance increasing crying episodes and sleep perturbed by more apneic spells, and can be involved in the sudden infant death syndrome [26, 33, 34].

Nose blocking also affects nasal chemosensation [35]. The disturbing effects of nasal closure during early development on the olfactory tract and function have been extensively demonstrated (mainly in the rat; e.g., [16]). Evidence for early structural alterations of the sense of smell due to nose-blocking is less well documented for humans [35, for review], but evidence shows that children's olfactory performance is significantly reduced [36, 37]. The clearest effect of adenoid-related nose blocking on olfaction is evidenced by the postoperative recuperation of children's awareness of food odours and their subsequent appreciation of eating [36]. Similar perceptual effects may operate in younger, preverbal infants when their nasal respiration and, hence, olfactory abilities are temporarily suppressed and then resume.

More or less chronic oral breathing has repeatedly been shown to induce a prolonged imbalance of oro-faciopharyngeal muscle activity. According to Moss [38], the muscular activity related to nasal breathing allows proper development of the craniofacial complex interacting with other functions such as mastication and swallowing [39]. This theory is based on the principle that facial growth depends on the functional activity of the different components of the head and neck region. For example, oral breathing imposed by adenoid hypertrophy has been suggested to explain the posterior rotation of the mandible [40]. Thus, oral breathing has been associated with increased mandibular inclination and changes in normal facial proportions, characterised by increased anterior lower facial height and decreased posterior facial height [41–43]. This induces the vertical axis of the facial skeleton to tend to develop excessively, resulting in an ogival palate (with consequences on dental occlusion) and dolichocephaly (or “long face syndrome”; [44, 45]) Similarly, experimentally induced nasal obstruction in young macaques (before and during pubertal development) induced permanent craniofacial deformities [46]. Long-term consequences of this developmental plasticity as a function of oral-breathing-induced craniofacial muscle mobilisation appear to be gender specific. For example, preschool boys suffering respiratory disorders during sleep presented higher anterior lower facial heights than girls [47].

To sum up, the shift from typical nose breathing to atypical mouth breathing in neonates and young infants illustrates how one function can have cascading effects on other functions to finally affect future form and functions. This forced change to oral respiration may impact all functions, from the most local [e.g., muscular exertion, craniofacial growth and functioning, chemosensory awareness, eating (sucking-swallowing articulation), and lower airway development] to the more general [sleep quality, temperamental traits, stress reactivity, and quality of life].

## 3. Multiple Impacts of Nasal Obstruction: The Rat as a Model

### 3.1. The Model: Methods and Outcome Measures

To further our understanding of nasal obstruction effects in general, we decided to investigate this problem in detail by using an animal model. For many reasons we chose the rat. To assess how momentarily perturbed nose breathing can affect oral competence as well as more general behavioural and physiological functions, an experimental technique of reversible bilateral nasal obstruction was developed that could be applied to newborn rats during their second week of life. After a first week of normal development, the pups underwent nasal closure for about 5 days to mimic the outbreak of a short blocking of nasal patency during early development of organic structures and functions. Nasal obstruction was performed on postnatal day (PND) 8 by bilaterally closing the external nares using an anaesthetic/surgical procedure currently applied to investigate the effects of closed nostrils on emerging olfactory function [48–51]. This procedure induced complete nasal closure between PND 8 and 12, with progressive reversal to unrestricted nasal airflow after PND 14. Different variables were measured on PND 9, that is, 24 h after the closure of both nostrils, on PND 15 to evaluate immediate and short-term effects, and up to PND 90 to evaluate long-term effects. The closed-nose (CN) pups were compared to sham-operated open-nose (ON) pups and to control (C) pups to evaluate oral competence and performance.

The following variables were quantified to assess the impact on organismal functioning, from the more local to the more general consequences: *feeding behaviour* [sucking behaviour of individual pups (nipple grasping ability, gastric content); maternal responses to pups (pup retrieval, presence in nest, and licking pups)]; *feeding-related structuresand functions* [oral activity; weight and myosin content of orofacial muscles; craniometric parameters]; *olfaction* [olfactory bulb size; nipple grasping performance; discrimination ability]; *metabolism-related consequences* [glycaemia, osmolality, hydration, and growth parameters]; *stress-related consequences* [weight of adrenal glands, plasmatic level of corticosterone, testosterone, and thyroid hormones].

### 3.2. Oral Competence: Functions—Oral Activation, Food Intake, and Feeding Interactions

Immediately after nasal blocking, the pups' inspiratory activity was redirected through the mouth as inferred from mouth-opening responses. This effect peaked on PND 11 (*n* = 23 mouth openings/min) to regress (*n* = 15 mouth openings/min) when nasal inspiration resumed on PND 14-15 [52]. In the same time, ON and C pups never exceeded 2 mouth openings/min. The fact that the respiratory effort is reassigned to the mouth may interfere with oral competence during suckling. Rat pups' oral performance was assessed directly by their capacity to grasp nipples orally after a period of separation from their dam, and by sucking success, directly evaluated by gastric milk content after a suckling trial. Significantly fewer CN pups than ON and C pups were able to attach to nipples between PND 9 and 15 [53]. In addition, during the days of enforced oral breathing, the sucking efficiency of CN pups that could suck was lower than that of ON and C pups, as shown by the significantly lower amounts of milk in their stomachs [54, 55]. Thus, nasal obstruction clearly interferes with normal sucking performance. First, pups appeared less proficient in attaching to nipples. Second, pups that did attach to nipples extracted milk less efficiently. When the nares had reopened by PND 15, the relative weights of milk taken became similar between groups for female pups, but were higher for CN male pups than for ON and C male pups [53]. So the impact of enforced oral breathing on pup feeding behaviour appeared to be restricted to the period of nose closure, but males expressed compensatory effects and ingested more milk when nasal respiration had been recuperated.

### 3.3. Oral Competence: Musculo- and Craniofacial Structures and Functions

The redirection, under experimental conditions, of newborn rats' breathing flow from the nose to the mouth recruits all reactive resources to ensure sufficient responsiveness of the organism. The new developmental situation imposed by blocking the nose alters the typical physiological constraints on local muscles and changes the mechanical stress on local bones. Muscles normally mobilised to fulfil respiration then incur extra work to keep the homeostasis of blood gases, but muscles involved in sucking and in social interactions are also recruited to maintain a satisfactory respiratory level.

**Figure 1:**
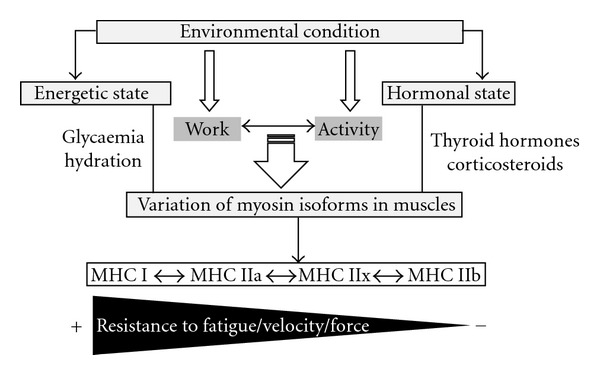
Diagram presenting the impact of environmental condition on myosin heavy chain (MHC) expression in adult skeletal muscles (I: slow; IIa: fast; IIx: fast; IIb: fast type fibres).

Skeletal muscles are composed of a combination of fibres classified on the basis of their contraction speeds and resistance to fatigue due to iterative stimulation, as slow twitch or fast twitch [55]. The contractile properties of muscles correlate with their myosin heavy chain (MHC) composition [56–58]. *Adult* skeletal muscles contain four major MHC isoforms, three being of the fast type (MHC IIa, IIx, and IIb) and one of the slow type (MHC I) [59]. MHC isoform expression determines muscle fibre contractile properties: fibres expressing MHC I generate less maximum specific force, slower shortening velocity, and greater resistance to fatigue than fibres expressing fast MHC isoforms (and among fast fibres, those expressing MHC IIx and IIb generate greater maximum specific force, faster shortening velocity, and lower resistance to fatigue than fibres expressing MHC IIa). Some MHC isoforms are specific to the perinatal period [60]: embryonic MHC (MHC_em)_ and neonatal MHC (MHC_neo_). Expression of the different myosin isoforms in skeletal muscles is developmentally regulated [61]. In fast-contracting rat muscles, MHC_neo_ replaces MHC_em_ to become the predominant type by 7–11 days after birth; subsequently MHC_neo_ is replaced by the fast adult isoforms [62–65]. Slow muscle fibres can develop through several pathways, but involve similar myosin isozyme transitions [63, 66]. The quality and quantity of expressed MHC isoforms of skeletal muscles are exceedingly plastic, and their fibre-type profiles can change in response to numerous factors, such as developmental stage, neuromuscular activity, physical mobilisation, and endocrine conditions [67–71]. These functional interactions are summarised in Figure 1.

The nasal obstruction episode in the present experimental series caused early changes in the structural/functional properties of rat pups' respiratory/orofacial muscles. Four muscles were targeted: the diaphragm, the *digastric anterior* (mandible depressor, opening the mouth), the *masseter superficialis* (mandible propulsor, closing the mouth), and the *levator nasolabialis* (involved in nasal flaring and sniffing). First, the relative weights of the last three muscles were considerably reduced (by 35, 33, and 66%, resp.) in pups following nasal obstruction [50, 72]. Further, during nasal obstruction, maturation of these muscles was enhanced in CN pups compared to ON and C pups. This is attested by the inversely correlated decrease of MHC_neo_ and increase of mature MHC isoforms in the diaphragm and orofacial muscles. This effect of oral inhalation was extremely rapid as the muscular differences among treatment groups could be seen within 24 h after obstruction.

During typical development, muscular MHC composition changes in an orderly fashion from embryonic to neonatal to adult fast/slow isoforms [67], and this change appears regulated in time (between 7–11 days after birth). Then MHC_neo_ decreases, disappearing entirely by PND 28 [68]. The short episode of nasal obstruction enforced here (between PND 9 and 11) clearly influenced these developmental changes, as the MHC_neo_ isoform increased normally in ON and C pups (Table 1), but not in CN pups [72]. Thus, nasal obstruction postponed maturational progression of the oral muscles that were recruited to work in respiration.

The early episode of nasal obstruction had *long-lasting effects* on the properties of the muscles considered in the facial-oral sphere, as these effects could be noted on PND 21 [50] and even on PND 90 [72]. The diaphragm of male rats undergoing CN treatment contained more of the MHC I (slow) isoform, and the target orofacial muscles contained more of the MHC IIa isoform at the expense of IIx and IIb isoforms (the most “fatigable”). The orofacial muscles involved in breathing showed an opposite profile, with decreased and increased expression of the MHC IIx isoform in the muscles involved, respectively, in closing and opening the mouth. Thus, the MHC phenotypes of rat pups exposed to a short episode of enforced oral breathing present plastic changes that appeared adaptive following the abrupt transition from nasal to oral breathing. Furthermore, following temporary forced nasal obstruction, the diaphragm and active sniffing muscles appeared consistently more resistant to fatigue in terms of MHC composition [72]. These phenotypic profile changes of MHC composition in CN rats' active sniffing muscles could be explained by decreased flaring and sniffing. The CN rat pups' mandibular muscle controlling mouth opening became more resistant to fatigue than the muscle controlling oral closing. Thus, although this result is explainable in terms of different controls of mouth opening versus closing muscles, temporarily forced oral breathing might produce long-lasting motor modifications in sucking behaviour associated with alterations of respiratory muscles' specific electromyographic activity.

Oral breathing in rat pups also caused long-term changes in craniofacial development. CN pups presented a symmetrical decrease of the vertical development of the nasomaxillary complex and of the longitudinal development of the skull-base [73]. Thus, an early nasal obstruction period was associated with delayed craniofacial development in both male and female pups. However, in the long run (namely, 90 days after nostril reopening), the craniofacial growth delay noted during the period of nasal obstruction did not persist in CN males in which the nasomaxillary complex and skull-base longitudinal axis has been reduced [73]. By contrast, only the longitudinal skull base of CN female pups remained somewhat shorter than that of controls as the animals grew older. Thus, the long-term osteologic effects of an early episode of oral breathing vary in relation to pups' sex.

### 3.4. Nasal Chemosensory Competence: Structure and Function

Nasal obstruction had a significant atrophic effect on the olfactory bulbs; bulbar weight of CN pups was about 30% less than that of control pups at PND 11 [73], and 50% less at PND 21 [50]. This bulbar reduction is relatable to decreased olfactory function as measured directly and indirectly. A test of odour-guided nipple attachment after a 2 h period of mother-offspring separation showed a perturbed response by CN pups (relative to controls) during the narial closure period (PND 9) and immediately after (PND 15), and the success in getting milk (gastric content) was accordingly reduced during the perturbation of olfaction [53, 54]. Further, in a paired choice-test comparing the odours of nest-sawdust and of clean sawdust, latency to choose was longer and duration of orientation towards the familiar nest odour was shorter for 9-day old CN pups than for control pups. By PND 15, when nasal respiration resumed, this difference was reduced due mainly to the return of nasal respiration in female CN pups [53]. Atrophy of the olfactory bulbs persisted in the long term (PND 90) in both sexes [73], although their exploratory and sniffing behaviours in a new environment became normal [74]. However, olfaction appeared to be permanently affected, as adult CN males exhibited impaired responses to sex-related odour cues [74].

### 3.5. General Systemic Responses (Viability, Homeostasis, Stress, and Behaviour)

Early exposure to an episode of nasal obstruction impacts on pup viability. Under our experimental conditions, mortality was nil in both control groups but reached 23% 72 h after narial closure in the CN group. On PND 21, the cumulative death rate reached 37% [52], suggesting that the consequences of perturbed oronasal function are protracted after the episode of nasal obstruction *per se*. This increased mortality rate is certainly multifactorial as all systemic regulations are concurrently affected by the respiratory mobilisation of the mouth. The *first* cause to be invoked is energetic depletion of the NC pups that were less competent in getting milk. *Second*, another immediate consequence of mouth breathing is air swallowing, especially during the process of sucking. Excess gas in the gastro-intestinal tract has been noted after nasal obstruction and related to the advent of necrosis and haemorrhages in the gut [75], in addition to diaphragmatic compression and paralysis of ileus leading to the arrest of intestinal transit [76] and increased risk of lethal perforation [77, 78]. A *third* cause involves the respiratory process itself. The effects of imposed oral breathing obviously affect blood gas parameters, leading to acute hypoxia, hypercapnia, and acidemia [79, 80], especially in neonates [81]. Adult rats' blood pH and O_2_ partial pressure are reduced 72 h after narial occlusion [82], leading to adverse changes in the homeostasis of blood gases. Nasal obstruction is also associated with an initial decrease in lung growth (PND 9–11), followed by recovery by PND 90 [74]. *Fourth*, NC rat pups' lessened oral competence caused by oral breathing may explain the small, but significant, decrease in plasma glycaemia on the first day of treatment, relatable to the reduced intake of milk reported above. *Fifth*, oral respiration increases evaporative loss, constituting an additional cause of body weight deficit and stress [83]. The significant increases in vasopressin release and plasma osmolality are indeed indicative of dehydration in CN pups [54]. Thus, any event enforcing oral breathing entails whole body dehydration [84]. *Sixth*, homeostasis is further imbalanced because of food-mediated maintenance of neonatal hormonal state. Thus, a few hours deprivation of mother's milk correlates with a significant reduction in thyroxin and an increase of plasma corticosterone levels [51, 53, 72, 85, 86]. Thyroid, renal, adrenal, and gonadal hormones play a key role in early development. An early deficiency in thyroid hormones disturbs brain development (specifically the olfactory system [87]) and delays the maturation of muscles (especially orofacial muscles) [88–90]. Vasopressin and corticotrophin-releasing hormone (CRH) both play a synergistic role in stimulating the release of adrenocorticotropic hormone (ACTH) [90], so vasopressin could possibly enhance the CRH effect during the first days of nasal obstruction-induced oral breathing. This “stress” reactivity might mediate response to nose-blocking surgery and/or dehydration induced by oral breathing [54]. The stress response induced by narial obstruction in 8-day-old rat pups is also evidenced by the hypertrophy of adrenal glands 72 h after treatment [72]. Adrenal hypertrophy is more marked in females (+68% in CN females and +29% in CN males, compared to controls) on PND 21 [51]. These effects did not persist over the long term (PND 90). An increase in plasma testosterone was observed during the nasal obstruction episode and on PND 90 [73]. This suggests that nasal obstruction *via* the olfactory bulb influences gonadotropin secretion that might be mediated by altering gonadal steroid feedback. *Seventh*, nose blocking affects the immune system by suppressing the proliferation of B-lymphocyte precursors [51]. Thymus weight was reduced only in CN females. The thymus is particularly sensitive to stress-associated glucocorticoids, which induce thymocyte apoptosis. *Eighth*, although not documented by our own experiments, nasal obstruction has far reaching consequences on biological rhythms. It can impair nocturnal sleep and induce diurnal lethargy [91–93]. We cannot exclude that it also induced biorhythmic maladaptation in rat pups, in terms either of hyporeactivity when they had to suck the nursing dam or of hyperactivity due to high corticosterone levels. *Finally*, a brief period of nasal obstruction affects mother-offspring interactions and decreases offspring's food intake [53]. Young rats' narial obstruction alters mother-pup interactions by reducing duration of retrieving and increasing pup licking by the dam. As already mentioned above, CN pups also showed lower mean duration of nursing and nipple attachment, which appeared related to difficulties in finding the nipple.

### 3.6. Summary and Limits of the Model

**Figure 2:**
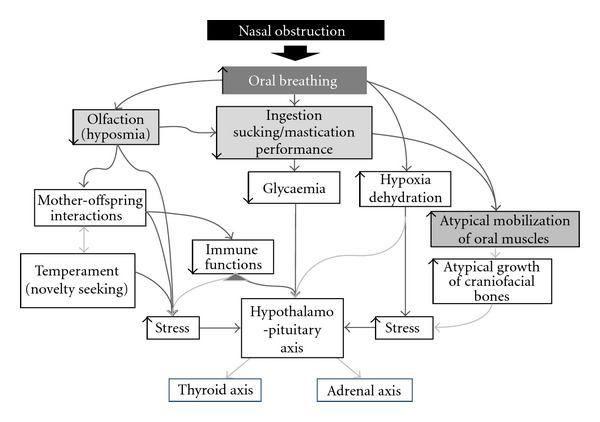
Representation of the main structures and processes involved in an episode of nasal obstruction in the neonate rat model.

The abrupt irruption of abnormal conditions of breathing in preweaning rat pups affects many local and general phenotypic traits over both short- and long-term developmental time scales. Oro-naso-facial growing structures and maturing functions are indeed shaped by the way they are solicited by their motor engagement in early respiration and ingestion. Thus, the oral and nasal pathways are tightly interdependent to ensure continued breathing when nasal occlusion occurs. However, this nasal defect-related oral compensation has immediate, short-term and long-term consequences (Figure 2).

The experimental results using the neonatal rat model of nasal occlusion may not be extrapolated in full to infants of other species. Thus, total obstruction of human infants' nasal airflow as in our model may be rare, as it is uncommon that both airways are completely blocked simultaneously [94]. However, premature infants initiate compensatory respiration through the mouth before complete occlusion of the nose, and O_2_ saturation is affected accordingly [95], suggesting that the negative impact on the oral function related to nasal obstruction may not require complete obstruction. Furthermore, under more natural physiological conditions, the incidence of nasal obstruction is probably more subtle and progressive, leading to more gradual adaptive responses [95]. Finally, the present neonatal rat model does not take into consideration the timing in which nasal obstruction occurs during early development. Postnatal development is indeed heterogeneous in relation to the various environmental challenges that neonatal organisms have to face, some periods and functions being potentially more sensitive than others. Nevertheless, if the above model of nasal occlusion has obvious limits to its generalisation, it reveals a complex pattern of interrelated effects involving all reactive abilities of neonatal and infantile organisms and raises important issues that can be generalised.

## 4. Discussion: Consequences for Human Neonates

What the above neonatal rat model teaches us, backed by extensive clinical observations in humans, is that the nose is more than a simple duct directing air to the lungs. From the very first breath (and perhaps before [13]), it also services sensory processes that are involved in the regulation of respiration (through trigeminal sensation) and of general behaviour mediated by the mouth (feeding motivation, orientation, and learning based on olfaction). At least in newborn and young mammals, the mouth has been emancipated from any involvement in respiration, leaving it reserved for ingestion, exploration, and communication. When incidental nasal obstruction occurs, all these functions are deferred in favour of maintaining air supply to the lungs. This change is far from benign as more than one-third of rat pups died from a 3-4-day nose obstruction in their second week of life. Such a high cost is fortunately not evident in humans. Neonatal and infantile organisms express considerable flexibility, as illustrated here by the outbreak of an abrupt shift to oral breathing in the neonatal rat model, there are functional limits and ceiling effects that need to be better understood in human infants.

A first major effect of this competition between respiration and ingestion at the mouth level is a reduced and disorganized sucking performance and a deprivation of sensory inputs to the developing olfactory tracts. It cannot be excluded that the dehydration incurred to oral and lingual mucosae by oral respiration may also affect gustatory abilities. Another major effect of the obligation to maintain breathing through the mouth is an altered oral competence in terms of muscular resistance and atypical shaping of the orofacial skeleton. Finally, early nasal obstruction or reduced patency has long-term consequences on biological rhythms and stress reactivity which, to our knowledge, have not yet been explored in human infants.

The other lesson derived from the neonatal rat model of nasal obstruction is that the organismal design is made of layers of adaptation, each with its own plasticity range and dynamics. Organisms can recruit various self-regulation processes to cope with challenges at different rates, and structures, forms, and organ compositions are induced by such challenges. Oral breathing (mouth opening) is the rapid response to nasal closure that also affects later the composition of the oral muscles mobilised by this new situation and the bones that support them. Then homeostasis of all endocrine systems is shifted towards maintaining energetic metabolism, hydration, growth, and stress response within limits. While some effects show rapid reversibility, others are slower to return to normal and others are nonreversible. Long-term consequences of nose blocking revealed by the rat pup model are related to the formation of the skull and oral structures, and to general reactivity. While the former long-term effects of nose obstruction have been described in human infants, the latter effect does not seem to have attracted much clinical attention. Finally, being exposed to the distress caused by a blocked nose may have variable consequences in relation to the subject's age and maturation. This is another point worthy of interest in human infants.

To summarise, organisms are integrated entities, and a function cannot be considered in isolation from the others. Thus, a change in oral function, even if it is only temporary, has repercussions on local and general functions. Such a change may be especially notable in more immature (namely, preterm) infants who must develop the skills needed to initiate oral feeding prior to progressing to coordinated sucking, breathing, and swallowing [96].

## Figures and Tables

**Table 1:** Distribution of myosin heavy chain (MHC) isoforms in selected oral (*digastric, masseter*) and nasal (*levator*) muscles in rats exposed to an early episode of forced oral breathing (CN group) and in control rats [74]. The different MHC isoforms were characterized on PND 11 and 90 (for key to the functions of the different MHC isoforms, see the text). Short-term nasal obstruction, that is forced oral breathing, leads to long-term orofacial muscle fibre adaptation. We observed increases in MHC neonatal and adult type I isoforms in muscles involved with oral breathing, *digastric,* and *masseter*, in CN group versus control on PND11. No changes were observed in the *levator* muscle involved with nasal breathing on PND 11. There are increases in MHC adult type IIb isoforms in muscle involved with oral breathing, *masseter*, and in muscle involved with nasal breathing, *levator*, in CN group versus control on PND 90. Values are given as percentages of total MHC and comparisons were then made using *t*-test with the Bonferroni correction.

MHC isoforms	emb	neo	I	IIa	IIx	IIb
On PND 11						
CN group						
Digastric	7	78^∗^	15^∗^	—	—	—
Masseter	9^∗^	91^∗^	—	—	—	—
Levator	14	86	—	—	—	—
Control group						
Digastric	6	85	9	—	—	—
Masseter	13	87	—	—	—	—
Levator	14	86	—	—	—	—

On PND 90						
CN group						
Digastric	—	—	—	20^∗^	44^∗^	37
Masseter	—	—	—	—	42^∗^	58^∗^
Levator	—	—	—	18^∗^	25^∗^	57^∗^
Control group						
Digastric	—	—	—	24	37	38
Masseter	—	—	—	—	48	52
Levator	—	—	—	1	31	68

^∗^Significantly different from control group at *t* = −10.37 to 26.03, *P* < 0.03 to <0.001.
